# Health Status, Intention to Seek Health Examination, and Participation in Health Education Among Taxi Drivers in Jinan, China

**DOI:** 10.5812/ircmj.13355

**Published:** 2014-04-05

**Authors:** Yan Yang, Xiao-sheng Fan, Cui-huan Tian, Wei Zhang, Jie Li, Shu-qing Li

**Affiliations:** 1 Health Examination Center, QiLu Hospital of Shandong University, Jinan, China; 2 Department of Cardiology People’s Hospital of LaiWu, LaiWu, China

**Keywords:** Health Status, Health Surveys, Occupational Disease, Health Education, Health Services Needs and Demand

## Abstract

**Background:**

Taxi drivers are exposed to various risk factors such as work overload, stress, an irregular diet, and a sedentary lifestyle, which make these individuals vulnerable to many diseases. This study was designed to assess the health status of this occupational group.

**Objectives:**

The objective was to explore the health status, the intention to seek health examination, and participation in health education among taxi drivers in Jinan, China.

**Patients and Methods:**

The sample-size was determined scientifically. The systematic sampling procedure was used for selecting the sample. Four hundred taxi drivers were randomly selected from several taxi companies in Jinan. In total, 396 valid questionnaires (from 370 males and 26 females) were returned. Health status, intention to seek health examination, and participation in health education were assessed by a self-designed questionnaire. Other personal information including sex, age, ethnicity, marital status, years of employment as a taxi driver, education level, and habits were also collected.

**Results:**

This survey revealed that 54.8% of taxi drivers reported illness in the last two weeks and 44.7% of participants reported chronic diseases. The prevalence rates of hypertension, diabetes mellitus, gastroenteritis, arthritis, and heart disease were 18.2%, 8.8%, 26%, 18.4%, and 4.8% of questioned taxi drivers, respectively. Significant self-reported symptoms included fatigue, waist and back pain, headache, dyspepsia, and dry throat affecting 49.7%, 26.2%, 23.5%, 26%, and 27% of participants, respectively. In total, 90.1% of subjects thought that it was necessary to receive a regular health examination. Only 17.9% of subjects had been given information about health education, and significantly, more than 87% of subjects who had been given information about health education reported that the information had been helpful.

**Conclusions:**

Taxi drivers’ health was poor in our survey. Thus, using health education interventions to improve knowledge and change in behaviors are necessary and effective programs that improve the health of individuals in this special occupational group are needed.

## 1. Background

In China, the “taxi” industry is an important component of the transportation industry that makes significant contributions to the city development. However, taxi drivers’ physical and mental health is poor. The average working time is 10.5 hours per day for 6.4 days per week, and these individuals spend an average of 3.5 hours waiting for passengers each day. Most taxi drivers have little education and a lack of economic, social, or human capital. Due to work overload, monotony, boredom, repetition, and the stress of coping with various traffic conditions, these drivers have a high risk of damaging the human biological clock rhythm and are at risk of fatigue, depression, tension, insomnia, and other diseases. Although most taxi drivers suffer from work-related diseases, to the best of our knowledge, few studies have explored the health status, the intention to seek health examination, and participation in health education among taxi drivers in China.

## 2. Objectives

For existing problems, we decided to conduct a study to explore the health status, the intention to seek health examination, and participation in health education among taxi drivers in Jinan, China.

## 3. Patients and Methods

### 3.1. Ethics Statement

This study used a descriptive design. The consent protocol and the procedure were approved by the Ethics Committee of Qilu Hospital of Shandong University, China. Participation in the study was completely voluntary and if participants did not wish to participate they would have the option of declining to answer specific questions or leaving the entire questionnaire blank. The survey was anonymous and the data were presented in an aggregated manner, hence, the participants' privacy and confidentiality would be firmly protected. All these policies were also printed in the front cover of the questionnaire. Informed written consent was received from all participants.

### 3.2. Demographic Characteristics

We obtained data from a cross-sectional survey conducted in Jinan, the capital of Shandong Province, China. We performed the survey from December 2012 to April 2013. Company-employed or self-employed taxi drivers were the targeted population. The sample-size was determined using the statistical cluster-sampling technique according to the formula: n = p (1-p) NZ^2 ^/ Nd^2^ + Z^2^ p (1-p). In total, 400 taxi drivers were randomly selected from several taxi companies in Jinan. Finally, 396 valid questionnaires were returned. The valid sample comprised 370 males and 26 females. A self-administered questionnaire was used to collect personal information including sex, age, ethnicity, marital status, years of employment as a taxi driver, and education level. Educational level was categorized into five categories: elementary school (i.e., people who attended up to six years of schooling), junior high school (i.e., 7-9 years of schooling), senior high school (i.e., 10-12 years of schooling), college, and university or higher (i.e., complete or incomplete post-secondary school).

### 3.3. Clinical Characteristics

Self-rated physical health was measured based on responses to the question “how would you rate your overall physical health?” using a five-point Likert-type scale, where 1 = excellent, 2 = good, 3 = fair, 4 = poor, and 5 = very poor. these five points are recommended by the World Health Organization (WHO) (1). On this scale, high scores indicate a better health status. Respondents were surveyed by asking “have you had any physical and mental discomfort during the past two weeks?” and “have you ever been diagnosed with chronic disease during the past 12 months?”. Those individuals who answered “yes” to the above questions were defined as having a clinical characteristic.

The history of disease was collected if an individual had been diagnosed with a chronic disease, such as hypertension, diabetes mellitus, heart disease, stroke, emphysema, ulcer, a liver condition, arthritis, weak/failing kidneys, asthma, cancer, or other medical conditions within 12 months prior to the survey. A chronic ailment is defined as an ailment that lasts or is expected to last for at least 12 months resulting in functional limitations or the need for ongoing medical services and includes disability (2). Non-physician-diagnosed chronic disease was not included. Physician-diagnosed chronic disease was further confirmed by asking about the location of diagnosis, including community clinics, county hospitals, city hospitals, provincial hospitals and military hospitals. The reported diseases were coded and classified using the disease classification scheme designed by China Ministry of Health for the survey.

Two widely used generic health-related quality-of-life measures are the EQ-5D and the 12-item short-form health survey (SF-12) (3-5). Based on them, we created a summary self-designed symptom questionnaire according to our actual situation. This questionnaire included questions about the most common symptoms. Other information about the intention to seek health examination and participation in health education was also recorded using our self-designed questionnaire.

### 3.4. Habits

To characterize health habits, we included data on the smoking status, alcohol consumption, irregular dietary habits, average working time per day, average sleeping time per day, and the frequency of physical activity. Smoking status was dichotomized as current smoker (≥ 1 filter per day) and nonsmoker. For physical activity assessment, exercise was grouped as “no” or “yes” for regular exercise, with people reporting "no planned walking" allocated into the “no” group, whereas any other planned exercise, including walking, was considered as eligible for the “yes” group.

### 3.5. Data Collection

Medical doctors and nurses at our center conducted the survey. Under the investigators’ guidance, the respondents completed the questionnaire themselves. For quality control, the completeness of the questionnaire was checked at the end of the survey. If there were missing data, individuals would be re-surveyed.

### 3.6. Statistical Analysis of Results

Proportions were employed to describe respondents’ basic characteristics and health status. Certain data were presented as the mean ± standard deviation (± SD). Because the number of females was small, we do not describe these individuals separately, and the P-value was not reported. SPSS version 10 (SPSS Inc, Chicago, Illinois, USA) was used for all statistical analyses. Ninety-five percent confidence interval (CI) was calculated for each variable.

## 4. Results

### 4.1. Basic Characteristics

The basic characteristic data of the respondents are presented in Table 1. Four hundred questionnaires were distributed, and 396 valid surveys were returned with a survey response rate of 99%. Participants' age ranged from 20 to 60 years, and the average age in the sample was 36.5±2.9 years. Most respondents were married (79.5%). Our survey revealed that more than 93% of taxi drivers were male (370 men), 70% were between 30 and 50 years of age, and 50% had junior high school education or below. The average number of years of employment as a taxi driver was 8.5 years.

**Table 1.** Basic Characteristics of Respondents ^a^

Characteristics	Results
**Sex**	
Men	370 (93.4)
Women	26 (6.6)
**Ethnicity**	
Minority Chinese	9 (2.2)
Han Chinese	387 (97.8)
**Marital status**	
Married (divorced and widowed included)	315 (79.5)
Unmarried	81 (20.5)
**Age group, y**	
20-30	69 (17.4)
30-40	161 (40.6)
40-50	117 (29.5)
50-60	49 (12.5)
**Education**	
Elementary school	59 (14.8)
Junior high school	136 (34.3)
Senior high school	188 (47.4)
College or above	13 (3.2)
**Years of employment as a taxi driver**	
1-5	52 (13.1)
5-10	203 (51.2)
≥ 10	141 (35.7)

^a^ Data are presented in No. (%).

### 4.2. Habits

Based on Table 2, of the respondents, approximately 76.5% were current smokers, 10.3% drank alcohol frequently, 66.9% had irregular diets, and 85% reported no regular exercise. The average working time was 10.5 hours per day and the average sleeping time was 6.9 hours per day.

**Table 2.** Habits ^a^

Characteristics	Results
**Currently smoking**	
Yes	297 (76.5)
No	99 (23.5)
**Drinking alcohol frequently (≥ 3 times per week)**	41 (10.3)
**Irregular diet**	265 (66.9)
**Working time, h**	10.5 ± 1.2
**Sleeping time, h**	6.9 ± 1.0
**Regular exercise in the last 6 months (≥ 3 times per week)**	
Yes	59 (14.8)
No	337 (85.2)

^a^ Data are presented in No. (%) or Mean ± SD.

### 4.3. Self-reported Chronic Diseases

As shown in Table 3 and 4, a total of 17 subjects (4.3%) rated their health as excellent, 123 (31%) as good, 166 (41.9%) as fair, 83 (20.9%) as poor, and 7 (1.7%) as very poor. Additionally, 54.8% reported illness in the last two weeks, and 44.7% reported chronic disease. The self-reported prevalence rates of hypertension, diabetes mellitus, gastroenteritis, arthritis, and heart disease were 18.2%, 8.8%, 26%, 18.4%, and 4.8%, respectively. Other reported chronic diseases included pulmonary problems (3.0%), liver problems (4.0%), renal problems (2.7%), infectious problems (4.5%), cancer (1.5%), and stroke (1.0%).

**Table 3.** Self-reported General Health Status

Characteristics	Number of People (%), (95% CI)
**Physical and emotional wellbeing**	
Excellent	17 (4.3), (2.3-6.3)
Good	123 (31), (26.4-35.6)
Fair	166 (41.9), (37-46.8)
Poor	83 (20.9), (16.9-24.9)
Very poor	7 (1.7), (0.4-3)
**Morbidity**	
Discomfort within 2 weeks	217 (54.8), (49.9-59.7)
Diagnosed with chronic disease during the past 12 months	189 (47.7), (42.8-52.6)
Infectious and parasitic disease	18 (4.5), (2.5-6.5)
Cancer	6 (1.5), (0.3-2.7)
Stroke	4 (1.0), (0.02-2)
Diabetes	35 (8.8), (6-11.6)
Heart disease	19 (4.8), (2.7-6.9)
Chronic pulmonary disease	12 (3.0), (1.3-4.7)
Hypertension	72 (18.2), (14.4-22)
Gastroenteritis	103 (26.0), (21.7-30.3)
Chronic liver disease	16 (4.0), (2.1-5.9)
Chronic renal disease	11 (2.7), (1.1-4.3)
Arthritis	73 (18.4), (14.6-22.2)

**Table 4.** Self-reported Symptoms

Characteristics	Number of People (%), (95% CI)
**Nervous system symptoms**	
Fatigue	197 (49.7), (44.8-54.6)
Headache	93 (23.5), (19.3-27.7)
Particular irritability	75 (18.9), (15-22.8)
Insomnia	62 (15.6), (12-19.2)
Dizziness	45 (11.3), (8.2-14.4)
Anxiety	57 (14.3), (10.9-17.7)
Dreaminess	61 (15.4), (11.8-19)
Hypomnesis	72 (18.1), (14.3-21.9)
**Digestive system symptoms**	
Anorexia	93 (23.5), (19.3-27.7)
Nausea and vomiting	43 (10.8), (7.7-13.9)
Dyspepsia	103 (26.0), (21.7-30.3)
Constipation	32 (8.1), (5.4-10.8)
Diarrhea	21 (5.3), (3.1-7.5)
**Renal system symptoms**	
Frequent micturition	87 (21.9), (17.8-26)
**Respiratory system symptoms**	
Cough	41 (10.3), (7.3-13.3)
Expectoration	39 (9.8), (6.9-12.7)
Sore throat	93 (23.5), (19.3-27.7)
Asthma	11 (2.7), (1.1-4.3)
Chest congestion	101 (25.5), (21.2-29.8)
Dry throat	107 (27), (22.6-31.4)
**Cardiovascular system symptoms**	
Heart palpitation	37 (9.3), (6.4-12.2)
**Joint and muscle system symptoms**	
Stiffness	53 (13.4), (10-16.8)
Waist and back pain	104 (26.2), (21.9-30.5)
Scapulohumeral periarthritis	27 (6.8), (4.3-9.3)

### 4.4. Self-reported Symptoms

The significant self-reported symptoms included fatigue, waist and back pain, headache, dyspepsia, and dry throat that affected 49.7%, 26.2%, 23.5%, 26%, and 27% of respondents, respectively.

### 4.5. Intention to Seek Health Examination and Participation in Health Education

Regarding education, as shown in Table 5, 90.1% of subjects thought that it was necessary to receive regular health examinations. Seventy-one (17.9%) subjects indicated that they had been given information about health education, and significantly, more than 87% subjects who had been given information about health education reported that the information had been helpful. In total, 43.6% of subjects indicated that lecture in the community was an important way to receive a health education message.

**Table 5.** Intention to Seek Health Examination and Participation in Health Education

Characteristics	Number of People (%), (95% CI)
**Health examination needs**	
Necessary	357 (90.1), (87.1-93)
Unnecessary	39 (9.9), (7-12.8)
**Received message about health education before **	71 (17.9), (14.1-21.7)
**Message was helpful**	62 (87.3), (84-90.6)
**Way of receiving health education message **	
Radio TV Internet Book	37 (52.1), (47.2-57)
Lecture in the community	31 (43.6), (38.7-48.5)
Doctor	23 (32.4), (27.8-37)
Other	5 (7), (4.5-9.5)

## 5. Discussion

Self-rated health (SRH) is based on a simple questionnaire on which people are asked to rate their own overall health. SRH is also termed “general health” because the phrase refers to an individual’s perception of his or her own health (6). Although SRH is a subjective consciousness about personal health rather than an objective measure of health, this factor has been reported to be a predictor of morbidity and mortality, even after controlling for other related factors (7). This study highlighted taxi drivers’ health status as follows. Most subjects rated their health positively and 35.3% rated their health as very good or good whereas 22.6% of subjects reported bad or very bad health. The study presents two main findings. First, the prevalence rate of hypertension (18.2%), lumbar muscle strain-related arthritis (18.4%), gastroenteritis (26%), and diabetes mellitus (8.8%) was high. Second, a significant number of subjects had symptoms such as headache, fatigue, anorexia, dyspepsia, dry throat, chest congestion, and waist and back pain.

Hypertension is one of the most important risk factors for stroke, coronary heart disease, and renal disease (8, 9). It has been hypothesized that by 2025, 29.2% of the world’s adult population would suffer from hypertension and most affected individuals would be found in developing countries (10). Our study indicates that the prevalence rate of hypertension among taxi drivers was 18.2% whereas the overall prevalence rate of hypertension in Chinese adults was 18.1% in 2004 (11). Taxi drivers experience decreased physical activity, hard work, job stress, and a disruption in normal sleep and waking patterns, which might explain the higher prevalence of hypertension among taxi drivers.

Gastritis is a serious medical problem for many people worldwide and untreated gastritis can cause a peptic ulcer or gastric cancer (12). Stress, an irregular lifestyle, certain eating habits, and sedentary work are associated with a higher prevalence of gastritis among taxi drivers.

Taxi drivers are constrained to a very limited space behind the wheel, and they have to assume driving postures without too much backward inclination to provide more room for passengers. Due to the long driving time (on average, 10 hours per day for 26 days per month), taxi drivers have to continuously work in stationary postures for a long period. Prolonged sitting behind the wheel can cause significant postural strains on the back muscles and the lumbar spine (13, 14). Taxi drivers are exposed to whole-body vibration on a daily basis, which results in joint pain, swelling, muscle stiffness, and back pain (15, 16). Our studies also confirmed these results. Furthermore, taxi drivers are exposed to off-gas air pollution and a hazardous work environment, which can cause respiratory system problems. Similar results were found in other studies (17).

It is well known that exercise and a healthy diet are determinants of better SRH (18). Numerous factors including stress, depression, bad habits (such as alcohol abuse, excessive smoking, staying up late, and a lack of exercise), and air pollution are correlated with the development and progression of many diseases especially cardiopathy, diabetes mellitus, and tumors (19). Promoting healthy lifestyles through health education is recognized as effective by many researchers (20-22). However, in our study, approximately 76.5% of the respondents were current smokers, 10.3% drank alcohol frequently, 66.9% had irregular diets, and 85% reported no regular exercise. Thus, using health education interventions to improve knowledge and change behavior seems necessary. Regarding health education needs, taxi drivers expressed a significant need as only 17.9% of our study participants had received a health education message, which indicated the need for a greater effort in this area.

A strength of the study is its representativeness for one nation, but several limitations to this study are worth noting. First, the study was a population-based cross-sectional survey, and the cross-sectional design hampered causality inference. Second, the measurements were self-reports and thus, were subjected to information and recall bias. Third, although quality of life is an important parameter in population health status, quality of life was not examined throughly in our study. While the study was conducted on a limited number of subjects, interestingly, the outcome revealed several facts about the health status of taxi drivers that would help in the promotion of their socioeconomic conditions. Based on the findings, effective programs are needed to improve the health of this special occupational group.
